# Don’t Forget What You Can’t See: A Case of Ocular Syphilis

**DOI:** 10.5811/westjem.2016.5.28933

**Published:** 2016-06-21

**Authors:** Monica I. Lee, Annie W.C. Lee, Sean M. Sumsion, Julie A. Gorchynski

**Affiliations:** * University of Texas, Health Science Center, San Antonio, Department of Emergency Medicine, San Antonio, Texas; † University of Texas, Health Science Center, San Antonio, Department of Ophthalmology, San Antonio, Texas

## Abstract

This case describes an emergency department (ED) presentation of ocular syphilis in a human immunodeficiency virus (HIV) infected patient. This is an unusual presentation of syphilis and one that emergency physicians should be aware of. The prevalence of syphilis has reached epidemic proportions since 2001 with occurrences primarily among men who have sex with men (MSM). This is a case of a 24-year-old male who presented to our ED with bilateral painless vision loss. The patient’s history and ED workup were notable for MSM, positive rapid plasmin reagin (RPR) and HIV tests and fundus exam consistent with ocular syphilis, specifically uveitis. Ocular manifestations of syphilis can present at any stage of syphilis. The 2010 Centers for Disease Control and Prevention guidelines now recommend that ocular syphilis be treated as neurosyphilis regardless of the lumbar puncture results. There is a paucity of emergency medicine literature on ocular syphilis. For emergency physicians it is important to be aware of iritis, uveitis, or chorioretinitis as ocular manifestations of neurosyphilis especially in this high-risk population and to obtain RPR and HIV tests in the ED to facilitate early diagnosis, and treatment and to prevent irreversible vision loss.

## BACKGROUND

Syphilis is known as the “great imitator” for its ability to infect any organ and cause diverse symptoms.^15^ Currently there is a re-emergence of syphilis for which the case count and rate is the highest recorded since 1995 in the United States.^1^,^3^,^4^,^9^ In 2000, the rates of syphilis were at an all-time low (2.2 cases per 100,000 persons) but by 2013 had more than doubled (5.5 cases per 100,000 persons).^1^ Syphilis is a common worldwide sexually transmitted infection and is notorious for facilitating the transmission of the human immunodeficiency virus (HIV). The incidences of syphilis were highest among women in age groups 25 to 29 years and 20 to 24 years in men, especially in men who have sex with men (MSM).^1^–^9^ Most of the case studies on ocular syphilis are isolated to the ophthalmology literature. As emergency physicians we should be aware and be able to recognize manifestations of ocular syphilis as a cause of painless vision loss and its high rate of coinfection with HIV. Painless bilateral loss of vision may be the only presenting symptom of syphilis, which can be observed in up to one-third of patients with neurosyphilis.^10^,^11^ Centers for Disease Control and Prevention (CDC) guidelines now recommend that any ocular manifestation of syphilis such as iritis, uveitis, or chorioretinitis, be treated as neurosyphilis, with a 14-day course of intravenous (IV) penicillin G, regardless of the stage of clinical presentation of syphilis or lumbar puncture (LP) results.^1^, ^3^, ^4^, ^5^ Delay or lack of treatment may lead to long-term neurologic complications such as blindness, paralysis, dementia, psychosis and stroke.

## CASE REPORT

A 24-year-old male presented to the emergency department (ED) with five days of acute painless progressive bilateral loss of vision without photophobia, discharge, trauma, or contact lens use. Review of systems was negative except for recent alopecia^18^,^19^ The patient was a sexually active homosexual male with a negative HIV test three months prior. Social history included marijuana, cocaine and methamphetamine use.

**Figure 1:**
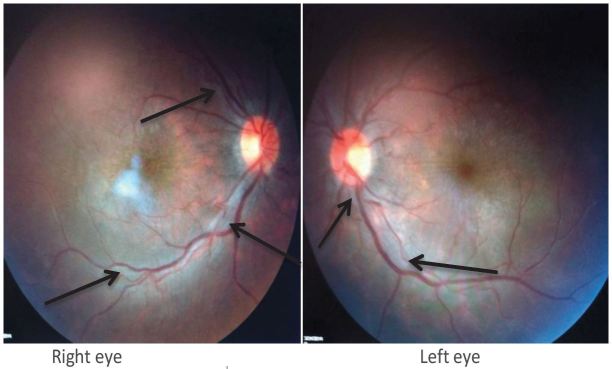
Vitritis Bilateral fundus photos. Vitreous haze, ½ +, retina is flat 360 degrees with arrows pointing to periphlebitis and diffuse homogenous retinal pallor.

**Figure 2:**
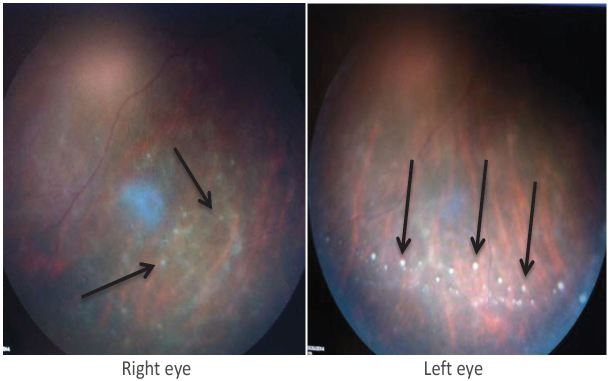
Uveitis Bilateral fundus photos: Inferior vitreous with arrows pointing to vitreous “ snowballs,” which represent aggregates of inflammatory cells at the level of the pre-retinal vitreous and peripheral retina.

ED vital signs reported a temperature of 98.7 degrees F, respiratory rate of 16 breaths per minute, heart rate of 90 beats per minute, blood pressure of 113/70 mmHg, 98% O2 saturation on room air, visual acuity of 20/600 bilaterally and intraocular pressures of 8 mmHg in the left eye and 10 mmHg in the right eye. Pupils were equal, round, reactive to light and accommodation and extra ocular movement intact, without Argyll Robertson (AR) pupils. Anterior chamber was clear, without conjunctival injection, foreign body, abrasion or ulceration. No afferent pupillary defect was appreciated. Ophthalmology was urgently consulted to evaluate the patient and perform a dilated fundus exam. Fundoscopy revealed bilateral vitritis (Figure 1) with scattered white tufts and globular white opacities inferiorly suggestive of posterior uveitis (Figure 2). These findings on the fundus exam are consistent with syphilis. The neurological exam was otherwise normal.

Given the patient’s social history and risk for sexually transmitted infections, specifically syphilis that can cause vision loss, and confirmation with ED rapid plasmin reagin (RPR) and HIV tests, ocular syphilis was high on the differential. Syphilitic posterior uveitis was the presumed diagnosis after the ED ophthalmology fundus exam and the first dose of a 14-day course of IV penicillin G was initiated in the ED with a medicine admission. Inpatient cerebral spinal fluid (CSF) results were a white blood cell (WBC) count of 33 cells/μL, red blood cell (RBC) count of 26 cells/μL, a protein level of 44 mg/dL, glucose 53 mg/dL, and a Venereal Disease Research Laboratory (VDRL) titer of 1:2. CSF ink stain, quantiferon gold, Lyme Ig, Bartonella Henselae, HBsAg, HCVAb were negative, as were bacterial and fungal cultures. The patient had an absolute CD4 count of 1347 cells/μL and a viral load of 52,900 c/mL HIV RNA. Magnetic resonance imaging of the brain and orbits were normal.

Current CDC guidelines recommend that any ocular manifestation of syphilis (irits, uveitis and choroidoretinitis) now be treated as neurosyphilis, regardless of the outcome of the LP.^3^–^5^ This is a departure from the past, where the clinical stage and CSF results (+VDRL, WBC cell count > 10 cells/μL, protein > 50 mg/dL) were the deciding factor for the treatment regimen for ocular syphilis. Since ocular manifestations can occur in secondary or tertiary syphilis the CSF results had previously been used to determine the presence of neurosyphilis. Current CDC guidelines recommend that all ocular manifestations of syphilis regardless of the stage of presentation or CSF results be treated as neurosyphilis with a 14-day course of IV penicillin G. An LP is still recommended since analysis provides additional evidence of other central nervous system (CNS) infections especially if the patient has a co-infection with HIV.^1^–^5^

## DISCUSSION

This is the case of a 24-year-old male who was assumed to be otherwise healthy presenting with painless vision loss due to uveitis as an ocular manifestation and presenting symptom of syphilis and concurrently found to be HIV positive. Ocular syphilis is a slow painless decrease in vision and there are no signs that are pathognomonic.^12^,^13^ A case report suggests a triad of headache, red eye or eye pain, and elevated erythrocyte sedimentation rate should prompt clinical suspicion for ocular syphilis.^12^ The AR pupil is highly specific for neurosyphilis, but as in this case it was not present. AR pupil has historically been associated with neurosyphilis but it has also been associated with diabetic retinopathy, multiple sclerosis, Wernicke’s encephalopathy, Dejerine-Sottas hypertrophic neuritis, Charcot-Marie-Tooth disease, herpes zoster, Lyme disease, sarcoidosis, midbrain lesions, and von Economo’s encephalitis.^12^,^14^ Other clinical findings of neurosyphilis may include third and sixth cranial nerve palsies, and visual field defects from brain involvement.^12^

Uveitis may manifest during secondary or tertiary syphilis, with iritis being the most common ocular finding in secondary syphilis.^5^,^7^,^8^,^15^ Syphilitic uveitis is the most common presentation of syphilis in older adults.^3^,^6^,^8^ The findings on the dilated eye exam were consistent with posterior uveitis, but non-specific for syphilis. However, in the setting of positive serum RPR and CSF leukocytosis and titers positive for VDRL confirmed the diagnosis of syphilitic uveitis, and other infectious and rheumatologic etiologies were concurrently excluded.^7^,^8^,^15^,^16^ The differential at the time of presentation included but was not limited to the following: Lyme disease, sarcoidosis, tuberculosis (TB), toxoplasmosis, toxocariasis, bartonella, brucellosis, herpes simplex virus, inflammatory bowel disease and rheumatologic conditions such as juvenile idiopathic arthritis and human leukocyte antigen (HLA)-B27-associated disease. 12

Uveitis is a state of inflammation involving the uvea (iris, ciliary body, choroid) or retina. This may be caused by autoimmune conditions, infections, or trauma, but up to 50% of cases are idiopathic. 8 Regardless of etiology, uveitis represents a breach in the blood-ocular barrier. Disruption of this barrier is the result of inflammation, a breakdown that allows neutrophils and other inflammatory mediators to incite the acute phase of uveitis. Both the anterior and posterior chambers, as well as the vitreous cavity, are susceptible to uveitis. Identification of the predominantly involved location can narrow the differential. Anterior uveitis (iritis, iridocyclitis), is primarily due to rheumatologic and idiopathic etiologies with herpes being the most common infectious cause. White blood cells invade the aqueous humor, with inflammatory changes often resulting in precipitation of inflammatory cells (neutrophils or macrophages) on the posterior cornea (keratic precipitates), as well as other iris changes. Intermediate uveitis or pars planitis, is rare and commonly idiopathic, but when present it is classically associated with multiple sclerosis. Posterior uveitis (choroiditis, retinitis, chorioretinitis, retina vasculitis) is more commonly associated with an infectious cause in up to 40% of reported cases with pathogens that include syphilis, toxoplasmosis, and cytomegalovirus. Panuveitis is the rarest form of uveitis that involves the entirety of the eye, which comprises only 10% of all cases of uveitis. Infectious causes include syphilis, TB and endophthalmitis, either bacterial or fungal.^8^,^15^,^16^

The presence of HIV may alter the presentation of syphilis, with possibly a more rapid progression to neurosyphilis.^1^ Syphilis is an important facilitator of HIV transmission with current reported co-infection rates of 50–70%.^5^,^7^,^17^ In 2001 the prevalence of syphilis was at its nadir, but infection rates have since reached their highest levels since 1995. A review of the current epidemiology of syphilis has described the syphilis epidemic occurring primarily among men, especially MSM.^1^–^4^,

Similar case reports were published primarily in the ophthalmology literature in the late 1990s and early 2000s but none recently. 20–22 Following are the CDC guidelines for the treatment regimen, which have been in place since 2010, and new recommendations for LP for ocular syphilis.

### Why Should Emergency Physicians Be Aware of This?

As emergency physicians we commonly see patients with vision complaints who have not had any prior evaluation by an ophthalmologist or a primary care physician. For this reason, ocular syphilis should be considered in all patients presenting to the ED with non-traumatic bilateral vision loss, especially among MSM. 2010 CDC guidelines recommend that any ocular manifestations of syphilis now be treated as neurosyphilis regardless at which stage it occurs with a 14-day course of IV Penicillin G. In addition, an LP is no longer required for CSF VDRL titers to determine the treatment regimen as previously had been required. However, an LP is still recommended for analysis of other concomitant CSF infections since syphilis has a high co-infection rate with HIV. With the current syphilis epidemic it is important for emergency physicians to recognize ocular manifestations of syphilis, and to order emergency department RPR and HIV tests in order to facilitate an urgent ophthalmology consultation, early diagnosis and treatment and to prevent permanent vision loss.
